# Tallness is associated with risk of testicular cancer: evidence for the nutrition hypothesis

**DOI:** 10.1038/sj.bjc.6604695

**Published:** 2008-09-30

**Authors:** K-P Dieckmann, J T Hartmann, J Classen, R Lüdde, M Diederichs, U Pichlmeier, for German Testicular Cancer Study Group

**Affiliations:** 1 Albertinen-Krankenhaus Hamburg, Klinik für Urologie, Hamburg, Germany; 2 Klinik für Hämatologie, Onkologie, Immunologie, Rheumatologie und Pneumologie, Südwestdeutsches Tumorzentrum, Universitätsklinik Tübingen, Tübingen, Germany; 3 Institut für Radio-Onkologie, St Vincentius Kliniken, Karlsruhe, Germany; 4 Institut für Wehrmedizinalstatistik und Berichtswesen der Bundeswehr, Andernach, Germany; 5 Institut für Medizinische Biometrie und Epidemiologie am Zentrum für Experimentelle Medizin, Universitätsklinikum Hamburg-Eppendorf, Hamburg, Germany

**Keywords:** testicular cancer, body size, childhood nutrition, seminoma, non-seminoma, BMI

## Abstract

The pathogenesis of testicular germ cell tumours (GCTs) is potentially influenced by high-energy nutrition during infancy. As adult height is a proxy for childhood nutrition, we investigated the role of nutrition in GCT pathogenesis by comparing stature of patients with healthy men. In a matched case–control study, 6415 patients with GCT were compared with healthy army conscripts (1:6 matching modus) with regard to height (cm) and body mass index (BMI; kg/m^2^). Statistical analysis involved tabulation of descriptive height measures and BMI. Conditional logistic regression models were used to quantify the association of GCT with height, with odds ratios (OR) adjusted for BMI. The literature was searched for studies on stature in GCT patients. Body size is significantly associated with risk of GCT, very tall men (>195 cm) having a GCT risk of OR=3.35 (95% confidence intervals (CI): 2.88–3.90; adjusted). Short stature is protective (OR=0.798; 95% CI: 0.68–0.93). Both histologic subgroups are associated with tallness. Of 16 previous reports, 7 were confirmative, 5 had null and 4 equivocal results. The association of stature with GCT risk accords with the nutrition hypothesis of GCT. This study expands the current view of GCT tumorigenesis by suggesting that high-calorie intake in childhood promotes GCT precursors originating *in utero*.

Adult stature results from a combination of genetic and environmental factors (Sinclair and Dangerfield, 1998). The most important environmental element impacting on childhood growth is nutrition (Gunnell *et al*, 2001). Attained adult height can thus be used as a biomarker for early childhood energy intake (Albanes *et al*, 1988). There is some indication for a positive association between adult height and overall risk of malignancy (Batty *et al*, 2006), although the data reported are inconsistent as yet (Giovannucci *et al*, 2004).

Testicular germ cell tumours (GCTs) are thought to originate *in utero*. Precursors of GCT, closely resembling primordial germ cells evolve in the primitive gonad, secondary to excess maternal oestrogen levels during embryogenesis (Dieckmann and Skakkebaek, 1999; Hoei-Hansen *et al*, 2005). Putatively, these precursors of malignancy may be promoted by factors acting during early childhood (Dieckmann and Pichlmeier, 2004), such as high-calorie nutrition. This assumption is mostly based on the observation that GCT incidence significantly decreased in post-war periods in Europe, when nutrition was mainly poor (Moller, 1993; Bray *et al*, 2006). Although, an association of GCT with tallness has often been postulated (Ehrengut *et al*, 1980), there are inconsistencies among the studies. In particular, methodological design was vastly different among the studies and some of the analyses were hampered by small sample size (Kleinteich and Marx, 1983; Whittemore *et al*, 1984). We have therefore, investigated the presumed association of GCT with height in a large patient sample and summarised previous data relating to this issue in the hope of resolving the question of tallness and GCT.

## Materials and methods

This investigation was designed as a matched case–control study with 228 participating institutions (appendix). Accrual started in 2002 and was terminated by the end of 2005. Cases treated during 1995–2005 were eligible (Dieckmann, 2007). Roughly 60% of the cases were enroled retrospectively. A total of 6415 patients were registered from all regions across Germany, ages ranging from 18 to 40 years. All were of Caucasian descent, and all had histologically confirmed testicular GCT, 58.4% non-seminoma and 41.6 % pure seminoma. Army conscripts of the German Army (Deutsche Bundeswehr) served as healthy controls. Data on national cohorts of conscripts born between 1965 and 1984 were supplied electronically by the Army Institute of Medical Statistics (Institut für Wehrmedizinalstatistik, Andernach), comprising 4 357 977 entries with anthropometric data of entire cohorts of young men aged 18–19 years examined by the German Army nation-wide during enrolment years 1985–2005.

The following anthropometric measures were recorded for each patient and control, respectively: Height (cm), weight (kg), and body mass index (BMI; kg/m^2^), as well as the exact date of birth. Histology of GCT was noted (pure seminoma or non-seminoma). Measurements for controls were those taken at military enrolment, whereas those for patients were mainly self-reported. As final adult height is usually attained at the age of 18–19 years, comparison of army conscripts with adult GCT patients is reasonable with respect to height. However, as body weight and BMI mostly continue to increase beyond age 19 (Mensink *et al*, 2005), these young army conscripts are no appropriate controls to compare with adult GCT patients. Accordingly, this analysis is limited to the role of adult height. Yet, data on BMI were recorded for purposes of adjustment in the course of statistical analysis.

For statistical comparison, a matching of patients with control persons according to exact date of birth was designed. In light of the minor variations regarding mean adult height among eastern and western regions in Germany, (Jaeger *et al*, 2001) we elected to employ a set of six individually matched control persons for each patient to overcome the potential confounding caused by regional variation. The final control population thus consisted of *n*=38 490 participants. In cases where more than six subjects were available for control among the original database, a random selection was performed.

### Statistical analysis

Matching and statistical analysis was accomplished by employing SAS software package (V.9. 1.3) on the Windows platform. First, a descriptive tabulation was performed regarding median height, 25th percentiles, 75th percentiles, as well as maximum and minimum height as found in the populations of GCT patients, controls, and in the subsets of seminoma and non-seminoma, respectively. For further detailed analysis, patients and controls were categorised with respect to height and BMI, respectively. Categories were <170, 170–174, 175–179, 180–184, 185–189, 190–194, ⩾195 with regard to height (cm), and <20, 20 to <25, 25 to <30, ⩾30 with respect to BMI (kg/m^2^). As the median height of German males aged 18–40 years currently ranges from 176 to 179 cm, the height category 175–179 cm was defined as the reference group. Conditional logistic regression models (Armitage *et al*, 2001) were calculated to obtain odds ratios (OR) and 95% confidence intervals (CIs) with and without adjustment for the potentially confounding factor, BMI. The Wald *χ*^2^-test was used to test the overall significance of each model parameter.

To identify previous controlled studies of adult height and testicular GCT, a literature survey was performed using electronic search systems (PubMed) and hand search of pertinent literature.

## Results

Analysis revealed that all statistical parameters of height that is, median, minimum, maximum, 25th percentile and 75th percentile are greater in patients than in controls (Table 1), whereas the subgroups of seminoma and non-seminoma, showed similar results (Table 1). With respect to height categories, the group ‘180–184 cm’ is the most frequent in both patients and controls, but those with taller height (i.e. >185 cm) are distinctly more frequent among cases than in controls, as with the subgroups of seminoma and non-seminoma. Conditional regression modelling with body height as the only parameter, given in Table 2, showed overall significant difference in relative frequencies of height categories between cases and controls (*P*<0.0001; Wald *χ*^2^-test). There is a significantly increasing risk of GCT with increasing height. As height may conceivably be interrelated with other anthropometric parameters, a descriptive analysis was performed stratifying by BMI categories. As shown in Table 3, patients exceed controls with respect to height in all of the BMI categories indicating that its confounding effect on GCT risk can only be low.

Conditional logistic regression adjusting for BMI confirmed the significant association of height with the risk of GCT (Table 2) and point estimates were almost equal in crude and adjusted models. It can be noted that shorter than average individuals (<170 cm) were at significantly decreased risk (OR=0.798; 95% CI: 0.688–0.926). Beyond average height, there is an incremental gain in risk with increasing categories of height. Very tall subjects (>195 cm) have a markedly increased risk (OR=3.35; 95% CI: 2.88–3.903). Homogeneous results were obtained after stratification into histological subtypes of GCT (Table 2). The findings were even more distinct when the lowest height category (<170 cm) was set as a reference in the logistic regression model. Then the highest category (>195 cm) showed an OR=4.199 (95% CI: 3.452–5.109) for GCT overall, and OR=4.045, and OR=4.345 for seminoma and non-seminoma, respectively. Sixteen previous reports on GCT in relation to height were identified, involving a total of 6602 cases.

## Discussion

Our study revealed a strongly significant association of adult height with the risk of testicular GCT. It is unique in three ways: first, it is by far the largest study to date; second, it included the highest risk ever reported; finally, there is a linear trend with increments of height whereas a distinctly decreased risk is found with short stature. The overall relative risk (OR) is 3.35 whereas previous OR ranged from 1.83 to 2.11 (Dieckmann and Pichlmeier, 2002; Swerdlow *et al*, 1989; McGlynn *et al*, 2007). It can be noted that GCT patients exceeded controls with respect to all the statistical measures of height, with both histological subgroups of GCT showing similar associations.

The results of the present evaluation are in line with seven previous studies (Kleinteich and Marx, 1983; Gallagher *et al*, 1995; Dieckmann and Pichlmeier, 2002; Rasmussen *et al*, 2003; Richiardi *et al*, 2003; Hardell *et al*, 2006; McGlynn *et al*, 2007) encompassing 3098 patients. Nine other studies had failed to demonstrate a significant correlation of body size with GCT risk (Whittemore *et al*, 1984; Swerdlow *et al*, 1989; Thune and Lund, 1994; UK Testicular Cancer Study Group, 1994; Davies *et al*, 1990, 1996; Petridou *et al*, 1997; Bjørge *et al*, 2006; Stang *et al*, 2006). However, it should be noted that three of these investigations found OR of borderline to be nonsignificant (Swerdlow *et al*, 1989; UK Testicular Cancer Study Group, 1994; Stang *et al*, 2006), and two disclosed significant associations with only one histological group of GCT (Bjørge *et al*, 2006; Stang *et al*, 2006). Importantly, there was no antithethical result. A formal meta-analysis of the above-mentioned studies is unfeasible because of the great differences in design, definition of referent standards and anthropometric categories. Overall, the huge present database in conjunction with the previous studies listed here provides compelling evidence for a virtually existing association of adult height with the risk of GCT.

This study has a number of strengths. The exceptional sample size leaves statistical chance effects at a minimum. Selection bias is unlikely because accrual of patients was performed nation-wide by 228 participating institutions across Germany. Similarly, the control population represented national standard because basically all young German men are enroled by the Federal Army. Confounding by age is ruled out because cases and controls were exactly matched by the day of birth. Conditional logistic regression models were with and without adjusting for BMI. As BMI is mainly associated with energy intake during adulthood, confounding of the present result by nutritional effects after adolescence is improbable. Yet, the study has certain weaknesses, such as controlling only for age and BMI. Anthropometric measures of patients were self-reported for the most part, whereas all of the controls had exactly measured values. As many men overestimate their own height (Blumer, 2007) there may be a systematic bias in the present results, but this is probably only modest. Overestimation of height usually does not exceed 1–2 cm, and mostly only men of short stature will do so. In this study, the proportions of categories with very tall men are distinctly higher among cases than controls, and too great for overestimation bias alone to explain. Moreover, there is some suggestion that overestimation of height is lower in patients than in healthy individuals (McGlynn *et al*, 2007). Individuals examined during military enrolment at age 18 could have developed GCT in later life. In that event, these individuals would represent grossly inappropriate controls. As, the life-time risk of a German male developing GCT is less than 1% (Dieckmann and Pichlmeier, 2004), the proportion of controls who might later become affected is far too low to represent a bias.

The association of tallness with testicular cancer risk lends much support to the nutrition hypothesis of GCT pathogenesis. In general, that theory implies an association of nutrition with GCT risk (McGlynn, 2001). As final adult height is largely determined during the first 2 years of life, it may be postulated from the present data that high-calorie nutrition after birth could have promoting or at least conserving effects on GCT precursor cells that are known to evolve during the intra-uterine period (Dieckmann and Pichlmeier, 2004). Direct evidence to support this hypothesis is difficult to establish. However, there are numerous indirect observations in accordance with the theory.

The main observation is the declining incidence rates of GCT in cohorts of men born during World War II or immediately thereafter (Moller, 1993; Bray *et al*, 2006). In that period malnutrition was common in Europe. When food supply improved some years later incidence rates resumed. The secular trend of increasing adult stature over time has been observed in many countries for many years. Similarly, the incidence rates of GCT have been increasing ever since (Garner *et al*, 2005; Bray *et al*, 2006; Huyghe *et al*, 2007). Whether or not these two observations are interrelated or just co-existent is unknown because the underlying reasons for both of these developments are obscure. However, what may point to a biological association is the fact that both of the developments could at least partly be explained by the increasing availability of food in the high-incidence nations over time. What also may suggest a true association is the observation that both trends, increasing GCT incidence rates (Pharris-Ciurej *et al*, 1999) and increasing adult stature (Jaeger *et al*, 2001), have potentially begun to abate.

Some decades ago, GCT had been found to be more frequent in ‘white-collar professions’ (Mustacchi and Millmore, 1976). That observation could also fit with the nutrition hypothesis because usually, white-collar professionals belonged to economically privileged classes and food supply was obviously sufficient. Currently, food deficiency is no longer a general problem in Western countries. Conversely, there is a clear trend towards higher prevalence of overweight and obesity, which is predominantly true in lower social classes. Accordingly, social gradients regarding GCT prevalence have levelled off (Pukkala and Weiderpass, 2002; Dieckmann and Pichlmeier, 2004). Overweight has also become a growing problem among Black American citizens (Hedley *et al*, 2004). In that subpopulation, GCT used to be a rather rare disease (Mustacchi and Millmore, 1976) and it is of particular note that the incidence has recently started to rise markedly (McGlynn *et al*, 2005).

Two ecological studies support the hypothesis, one showing a correlation between high-fat intake and the incidence of GCT (Armstrong and Doll, 1975) and the other reporting an association of dairy products specifically cheese, with the incidence of testicular cancer (Ganmaa *et al*, 2002). It is to be noted that, a Swedish cohort study reported an increased risk of GCT for individuals having high cholesterol levels at the edge of adulthood (Wirehn *et al*, 2005). As serum cholesterol levels may serve as a proxy for high-energy intake, it may be speculated that high fat consumption during childhood may have a bearing on GCT pathogenesis. Four case–control studies supported the connection of GCT risk with dairy food consumption (Davies *et al*, 1996; Sigurdson *et al*, 1999; Garner *et al*, 2003; Stang *et al*, 2006) whereas three others did not (Bonner *et al*, 2002; Walcott *et al*, 2002; McGlynn *et al*, 2007). Overall, direct evidence by controlled studies to support the nutrition hypothesis of GCT is scarce; however, the bulk of indirect evidence is clearly in favour of the hypothesis.

There is now sufficient evidence to acknowledge tallness as a recognised risk factor of testicular GCT. The underlying biological mechanisms linking tallness and GCT risk are less clear. However, the nutrition hypothesis outlined herein would provide an appealing explanation. According to that hypothesis, high-calorie intake during early childhood could advance length growth and promote GCT precursor cells at the same time. This hypothesis would thus complement and refine the current view of GCT pathogenesis that involves the development of GCT precursor cells during intra-uterine life.

## Figures and Tables

**Table 1:** Descriptive statistical analysis of height measures (cm)

	**n**	**Min.**	**25th pctl**	**Median**	**75th pctl**	**Max.**
GCT	6415	150	177	181	186	211
Controls	38 490	145	175	180	184	210
						
Seminoma	2667	150	177	182	186	207
Controls	16 002	145	175	180	184	210
						
Non-seminoma	3748	150	177	181	186	211
Controls	22 488	145	175	180	184	208

GCT=germ cell tumour; min.=minimum height; max.=maximum height; pctl=percentile.

**Table 2:** Conditional logistic regression with body height as covariate – unadjusted and adjusted for BMI, stratified for all GCT cases, seminoma, and non-seminoma cases

	**All GCT cases**				**Seminoma cases only**				**Non-seminoma cases only**			
	**Unadjusted for BMI**		**Adjusted**		**Unadjusted**		**Adjusted**		**Unadjusted**		**Adjusted**	
**Height (cm)** a	**OR**	**95% CI**	**OR**	**95% CI**	**OR**	**95% CI**	**OR**	**95% CI**	**OR**	**95% CI**	**OR**	**95% CI**
<170	0.815	0.705–0.941	0.798	0.688–0.926	0.882	0.707–1.100	0.842	0.668–1.059	0.769	0.635–0.931	0.764	0.629–0.929
												
170–174	0.955	0.868–1.050	0.928	0.841–1.023	0.943	0.811–1.095	0.895	0.766–1.047	0.963	0.852–1.089	0.946	0.834–1.072
												
175–179	1.0		1.0		1.0		1.0		1.0		1.0	
												
180–184	1.408	1.306–1.519	1.387	1.283–1.499	1.417	1.260–1.593	1.395	1.235–1.576	1.403	1.271–1.548	1.380	1.248–1.526
												
185–189	1.542	1.419–1.676	1.575	1.445–1.717	1.597	1.403–1.817	1.641	1.433–1.878	1.505	1.349–1.678	1.531	1.369–1.712
												
190–194	1.979	1.781–2.199	2.069	1.854–2.309	2.094	1.776–2.469	2.232	1.876–2.656	1.903	1.658–2.184	1.970	1.710–2.270
												
⩾195	3.194	2.764–3.691	3.353	2.880–3.903	3.201	2.570–3.987	3.404	2.691–4.306	3.192	2.634–3.868	3.322	2.720–4.056

CI=confidence interval; GCT=germ cell tumour; OR=odds ratio.

a The overall effect of body height is statistically significant in both models, unadjusted and adjusted for BMI (Wald *χ*^2^-test *P*<0.0001).

**Table 3:** Quantitative distribution of body height stratified by BMI category

**BMI (kg/m^2^)**	**Pop.**	**n**	**Min. (cm)**	**25th pctl (cm)**	**Median (cm)**	**75th pctl (cm)**	**Max. (cm)**
< 20	Controls	7880	157	176	180	185	210
	Cases	436	155	177	182	187	202
							
20 to <25	Controls	23 812	148	175	180	184	210
	Cases	3300	150	177	181	186	211
							
25 to <30	Controls	5327	156	175	180	184	208
	Cases	2062	150	177	181	186	204
							
30+	Controls	1471	145	175	179	184	203
	Cases	617	154	176	180	186	206

BMI=body mass index; max.=maximum; min.=minimum; pctl=percentile; pop.=population.
